# The Antioxidant Power of a Diet May Improve the Quality of Life of Young Women with Acne Vulgaris

**DOI:** 10.3390/nu16091270

**Published:** 2024-04-25

**Authors:** Kinga Zujko-Kowalska, Beata Jankowska, Małgorzata Elżbieta Zujko

**Affiliations:** 1 Department of Cardiology and Internal Medicine with Cardiac Intensive Care Unit, Medical University of Białystok, M. Skłodowskiej-Curie 24a, 15-276 Białystok, Poland; kinga.zujko-kowalska@sd.umb.edu.pl; 2 Department of Cosmetology, Faculty of Health Sciences, University of Łomża, Akademicka 14, 18-400 Łomża, Poland; bjankowska@al.edu.pl; 3 Department of Food Biotechnology, Faculty of Health Sciences, Medical University of Białystok, Szpitalna 37, 15-295 Białystok, Poland

**Keywords:** dietary antioxidants, acne vulgaris, quality of life

## Abstract

Acne vulgaris (AV) significantly reduces the quality of life (QoL) of young people, so it is important to look for factors that can improve their QoL. The aim of this study was to assess the relationship between dietary antioxidants measured using the new DAQI index and QoL measured using standardized tests. The DAQI included the following elements: antioxidant vitamins, minerals, carotenoids, polyphenols, phytosterols, lignans, and the total antioxidant capacity of the diet. The study involved 165 young women with AV, mainly students. A self-report survey was used to collect basic data on their sociodemographic status, anthropometric information, and lifestyle. The energy value of the diet and the content of vitamins, minerals, and carotenoids with antioxidant activity in the diet were estimated using 3-day food diaries and the Diet 6.0 program. The antioxidant potential of the diet and the content of polyphenols, phytosterols, lignans, and selenium were calculated based on the consumption of individual food products and available databases. The results of this study showed that the QoL of the young women with AV was impaired. However, greater adherence to an antioxidant diet reduces the risk of AV impact on the QoL by approximately 30–32% and the risk of depression by 33%. The DAQI may be used as a new indicator of diet quality in acne vulgaris.

## 1. Introduction

Acne vulgaris (AV) is a chronic skin disease that affects over 9% of the world’s population and over 5% of the European population. It appears mainly during adolescence (85%) but often persists into adulthood [1,2,3]. AV usually presents with comedones, papules, pustules, nodules, and scars, which most often occur on the face [4]. The presence of acne, especially on exposed body parts such as the face, can cause a significant psychological burden, negatively impacting quality of life (QoL) [5]. Therefore, multi-aspect, holistic patient care is important, taking into account both the physical and psychological consequences of acne [6]. 

The pathophysiology of acne depends on many factors, the most important of which are environmental, genetic, hormonal, and inflammatory factors, as well as gender, age, the presence of acne in the family, a sedentary lifestyle, and eating habits [7]. Many studies have confirmed that health-promoting dietary patterns such as the Mediterranean diet, vegetarian diet, low glycemic index of a diet, dietary probiotics, prebiotics, fiber, and polyunsaturated fatty acids are associated with a reduction in acne lesions. Meanwhile, ultra-processed foods that are rich in saturated fatty acids, sugar, and milk may intensify acne lesions [8,9,10]. Eating habits have been shown to be associated with the QoL in young people with AV [11]. However, although the role of diet in the development and severity of acne has been widely studied, the findings still remain controversial. 

In the pathogenesis of AV, oxidative stress plays an important role, defined as a disturbed oxidation–antioxidant balance. A small, physiological amount of reactive oxygen species is needed for the proper functioning of the immune system. However, their overproduction leads to cell damage and, consequently, to skin diseases [12]. Exogenous antioxidants contained in the diet support the action of endogenous antioxidants in eliminating the effects of oxidative stress [13,14,15]. Some authors have shown that selected dietary antioxidants (vitamins, minerals, polyphenols) have a beneficial effect on the prevention and treatment of AV [16,17]. However, it is worth noting that the entire diet contains a variety of antioxidants with complementary effects. Therefore, in this study, we used a new indicator of the antioxidant quality of the diet.

This study presents for the first time the relationship between the DAQI (dietary antioxidant quality index) and the QoL (measured by standardized questionnaires: SWLS, DLQI, CADI and BDI) of young women with AV. 

## 2. Materials and Methods

### 2.1. Study Population and Data Collection

The study group included 165 young women with AV. All the subjects agreed to participate in this study. The inclusion criteria were as follows: age 18–35 years, presence of acne vulgaris, a diet with a caloric value 500–5000 kcal. The exclusion criteria were as follows: age below 18 years and above 35 years, a diet with a caloric value below 500 kcal and above 5000 kcal, the presence of other skin diseases (rosacea, psoriasis, eczema). Acne was classified by a dermatologist and cosmetologist according to the GAGS (Global Acne Grading Scale) [18,19].

A self-report survey was used to collect baseline data on the subjects’ sociodemographic status, anthropometric information, lifestyle, and acne. On this basis, data were collected regarding body weight, height, level of education, work/study, marital status, smoking, alcohol consumption, and information about acne. The commonly known formula [body weight (kg)/height (m)^2^] was used to calculate BMI (body mass index). The level of physical activity was assessed using the IPAQ (International Physical Activity Questionnaire) and classified as a low, moderate, and high level of physical activity [20].

The tests assessing the QoL are presented in Table 1. 

Food intake was assessed based on 3-day food diaries (two randomly selected weekdays and one weekend day). The participants recorded all the foods and drinks that they consumed during each day of the study. Then, the results from the food diaries were calculated using the Diet 6.0 program. On this basis, the energy value and the content of some vitamins and minerals in the diet were estimated. The selenium, polyphenols, phytosterols, lignans, and antioxidant potential of the diet are not included in this computer program, so we used available databases [26]. 

### 2.2. Antioxidant Power of a Diet

The DAQI (dietary antioxidant quality index) described in our previous study was used to assess the antioxidant power of the diet [26]. The DAQI scale consists of the following dietary elements: β-carotene, vitamin C, vitamin E, selenium, iron, zinc, copper, manganese, dietary antioxidant capacity, lignans, polyphenols, and phytosterols. Due to the fact that diets contain a variety of antioxidants, we have developed an index of 12 dietary antioxidants. The minerals included in this index are components of antioxidant enzymes (glutathione peroxidase, superoxide dismutase, and catalase).

The cut-off values of the dietary components are presented in Table 2. The RDA (recommended dietary allowances) for vitamin C, iron, zinc, copper, selenium, and AI (adequate intake) for vitamin E and manganese were adopted in accordance with the nutrition standards of the Polish population [27]. The values that were ≥90% RDA/AI were accepted as normal intake, and values < 90% RDA/AI were classified as abnormal intake. For the remaining dietary components for which no nutrition standards were defined (β-carotene, lignans, phytosterols, polyphenols, and dietary antioxidant potential), values above and below the median were adopted after conversion to a 1000 kcal diet.

### 2.3. Statistical Analysis

Statistical analyses were performed using IBM SPSS Statistics v. 27.0 software (SPSS Inc., Chicago, IL, USA), and a *p* value of less than 0.05 was accepted as significant. The continuous variables were described as means (M) and standard deviations (SD), whereas the categorical variables were presented as counts (N) with percentages (%). We used the following statistic tests: Mann–Whitney–Wilcoxon, Kruskal–Wallis, and Pearson’s chi-squared. The participants were divided into three groups according to their DAQI tertiles. To assess the association between QoL and DAQI tertiles, multivariate logistic regression models were used. The data are illustrated as odds ratios (ORs) and 95% confidence intervals (CIs). Three models were developed: Model 1—crude data, Model 2—data adjusted for age and daily energy intake; Model 3—data adjusted for variables in Model 2 and BMI, level of physical activity, smoking, and severity of acne.

## 3. Results

Table 3 showed the general characteristics of the study population (young women with acne vulgaris).

The mean age of the women was 23.65 ± 6.24 (range: 18–35) years. In the study group, 88% were single, 76% were learning, and 65% had middle level of education. Most of the participants had an average BMI (75%), moderate physical activity (55%), and occasionally drank alcohol (58%). However, a large group of women (44%) had a low level of physical activity, 26% smoked cigarettes, 42% drank alcohol once a week or more often, and 14% were overweight. Over 52% of the women had struggled with acne vulgaris for 2 to 5 years, and 27% for over 5 years. Only 20% of the women had acne for less than 2 years. Among the participants, 37% had mild and 54% moderate acne severity. Severe acne affected a small percentage of the study population (9%).

The results of the QoL assessment tests are shown in Table 4. The mean CADI value was 8.14 ± 4.25, indicating a moderate impairment in QoL. In this test, 41% of women declared moderate impairment, 56% no or mild impairment, and only 3% severe impairment.

The mean DLQI value was 9.52 ± 4.36, which confirmed the moderate effect of acne on the QoL. In the DLQI test, 47% of the participants indicated a moderate effect of acne on their QoL, 42% no effect or a small effect, while 11% reported a very large or extremely large effect of acne on their QoL.

The mean SWLS value was average (20.32 ± 4.56). In the SWLS test, 40% of the women had an average life satisfaction, but 28% had a low life satisfaction, and 32% high a life satisfaction.

The mean BDI value indicated the absence of depression in the study group (9.75 ± 6.31). No depression was detected in 70% of the women. However, 25% of the participants had mild depression and 5% had moderate or severe depression. 

**Figure 1:**
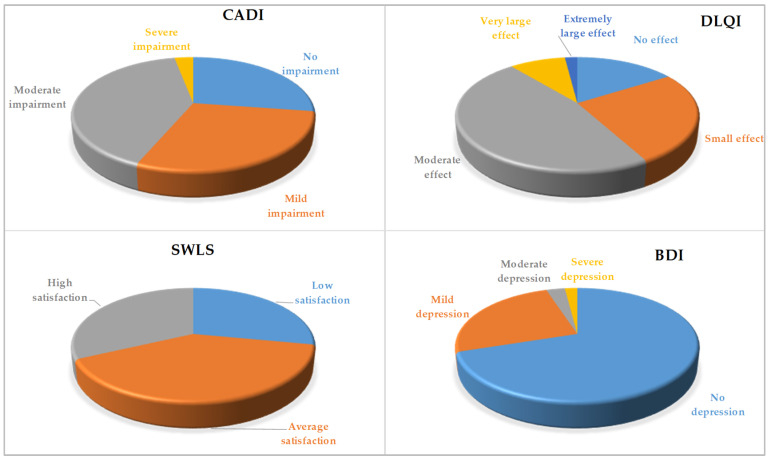
Quality of life measured using CADI, DLQI, SWLS, and BDI questionnaires.

Figure 1 illustrates the results of the CADI, DLQI, SWLS, and BDI measurements.

The calculation results of the DAQI are presented in Table 5. The obtained results indicate the average antioxidant power of the diet. Most of the participants consumed vitamins and minerals in amounts that were lower than recommended. Similarly, the dietary intake of phytosterols, polyphenols, and lignans and the antioxidant potential of the diet of most of the women were below the median. Only approximately 50% of the subjects consumed β-carotene above the median. 

Table 6 illustrates the relationships between the study population characteristics and DAQI tertiles. It was found that DAQI was significantly related to age (*p* = 0.011), BMI (*p* = 0.009), energy of diet (*p* < 0.001), physical activity (*p* < 0.001), smoking (*p* < 0.001), and the severity of acne lesions (*p* = 0.024). There was no association between DAQI and education, working status, marital status, and alcohol intake. 

The relationships between DAQI and QoL are presented in Table 7. In Models 1, 2, and 3, the highest tertiles of DAQI compared to the first tertiles reduced the risk of moderate and severe QoL impairment by approximately 30%, as measured by the CADI score (Model 3: OR = 0, 70, 95% CI: 0.42–0.98) and approximately 32% of the moderate, very large, and extremely large impact of acne on the QoL measured based on the DLQI scale (Model 3: OR = 0.68, 95% CI: 0.46 –0.96). Moreover, the highest DAQI tertiles reduced the risk of depression by approximately 33% (Model 3: OR = 0.67, 95% CI: 0.42–0.97). No association was found for SWLS and DAQI.

**Figure 2:**
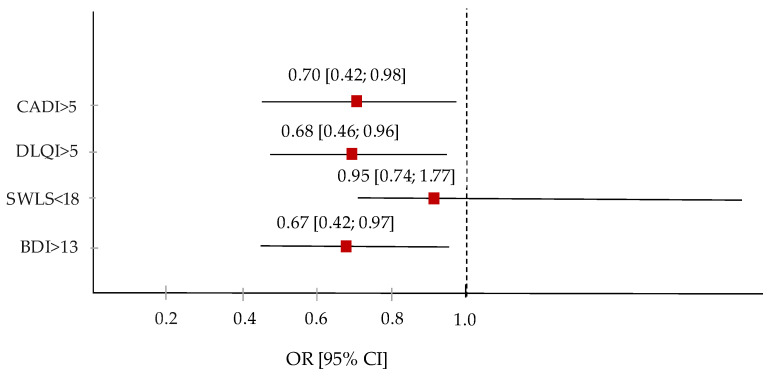
Odds ratio of reduced impact of AV on QoL with increasing DAQI. The red squares and horizontal lines represent the odds ratio (OR) and 95% CI, and the dashed line shows Tecyl 1.

Figure 2 illustrates the odds ratio of the reduced impact of acne vulgaris on quality of life with increasing antioxidant diet quality index. 

## 4. Discussion

Acne vulgaris is a serious social problem that most often affects young people and significantly reduces their quality of life. Therefore, identifying factors that can improve the QoL of people with AV is an important element of modern medicine [28]. It has been proven that only holistic patient care, taking into account physical and emotional aspects, can bring beneficial results [6]. In this study, AV had a moderate impact on the participants’ QoL, as measured by the CADI and DLQI questionnaires. Additionally, the respondents had an average satisfaction with their life as measured by the SWLS questionnaire. Moreover, almost one-third of the subjects showed depression, mostly mild, as measured by the BDI questionnaire. This study included young women aged 18–35, mainly with mild and moderate acne, for whom the condition of the skin is very important. Some authors have shown that in a group of young people, a greater reduction in QoL due to acne was associated with the severity of acne and the female gender [29]. 

In the prevention and treatment of AV, an important role is played by modifiable lifestyle factors, including an average BMI, moderate physical activity, avoiding smoking and drinking alcohol, and proper eating habits. In our study, a large group of respondents led an unhealthy lifestyle due to smoking, alcohol drinking, and low physical activity. Moreover, most of the participants’ intake of dietary antioxidants (vitamins and minerals) was below the dietary recommendations. 

Some authors have shown that a higher BMI is associated with the occurrence of AV due to a higher glycemic load and higher levels of androgens, which cause greater sebum production [30]. However, in our study, as many as three quarters of the participants had an average BMI, and only 14% were overweight.

Smoking causes significant changes in skin microcirculation, keratinocytes, and the synthesis of collagen and elastin, and it also delays wound healing and accelerates skin aging [31]. Moreover, it causes peroxidation of skin lipids [32]. In this study, a quarter of the subjects smoked cigarettes.

The results of other studies have shown that the habit of drinking alcohol is a predisposing factor to the occurrence of acne [33]. Alcohol increases the production of pro-inflammatory cytokines and suppresses the immune system, which leads to changes in the skin microbiome [34]. In our study, almost half of the young women reported drinking alcohol once a week or more often.

It is well known that regular, moderate physical activity has a positive effect on overall health and improves the condition of the skin, its ability to retain moisture, and prevents future skin problems [35]. In our study, almost half of the participants had a low level of physical activity.

The relationship between diet and skin conditions has been studied for a long time. Diet influences the AV through the immune and hormonal system, gut microbiota, and carbohydrate and lipid metabolism. Epidemiological studies indicate that the prevalence of AV is lower in developing countries compared to highly developed countries. The typical Western diet, rich in ultra-processed foods with sugar, saturated fatty acids, and food additives (chemical preservatives, synthetic colorants, flavor enhancers) with a high glycemic index and being poor in polyunsaturated fatty acids, fiber, water, vitamins, and minerals, can predispose individuals to the development of AV [10,36,37]. Many studies have shown that an improper diet has a pro-inflammatory effect and contributes to the disruption of the diversity of the gut microbiome, influencing the development of chronic diseases including dermatological diseases [38,39,40]. Healthy dietary patterns, such as the Mediterranean diet, have been shown to have a protective role in the pathogenesis of acne vulgaris [41,42,43,44]. However, some studies do not confirm this relationship [45]. 

This study examined for the first time the relationship between the dietary antioxidant quality index and the quality of life of patients with acne vulgaris. The mean DAQI was found to be 8.2 ± 4.8, indicating a moderate antioxidant power of the diet. People with a higher DAQI had a healthier lifestyle, i.e., they had a lower BMI, moderate physical activity, and did not smoke cigarettes. Moreover, a higher DAQI was associated with lower AV severity. In fully adjusted models, regardless of confounding variables, the highest tertiles of DAQI reduced the risk of AV impact on QoL by approximately 30–33%.

The new DAQI was developed by taking into account various antioxidants such as dietary vitamins, carotenoids, minerals, polyphenols, phytosterols, lignans, and the total antioxidant capacity of the diet. Previous research by other authors has shown the beneficial effects of individual antioxidants on the condition of the skin. It was found that plasma levels of vitamins E, A, selenium, and zinc were lower in AV patients compared to the control group [46,47,48]. Moreover, polyphenols inhibited sebum production, lipogenesis, and proliferation of sebaceous cells and had a bactericidal effect on *P. acnes* [49,50]. Vitamin C was also widely used in skin diseases. It prevented photoaging, discoloration, and tissue inflammation, supported tissue healing, and stimulated collagen production [51]. Oxidative stress in AV is manifested by changes in the activity of antioxidant enzymes, whose cofactors are minerals such as zinc, selenium, copper, manganese, and iron [52]. During oxidative stress, the level of antioxidant systems is exceeded, which causes a change in the function and molecular composition of keratinocytes and sebaceous cells and contributes to the progression of the inflammatory process [53].

The pathogenesis of acne vulgaris is not fully known. However, the determination of oxidative stress biomarkers, especially lipid peroxidation in acne patients, confirms the important role of oxidative stress. Inflammation caused by oxidative stress of the pilosebaceous unit and oxidation of sebum initiates the development of acne [54]. A well-composed diet is a source of various antioxidants (vitamins, minerals, polyphenols) that may support the treatment of acne and improve quality of life [55]. 

### Limitations

This study has some limitations. First, food intake was assessed based on 3-day food diaries, which did not take into account usual eating habits. Second, the available databases on dietary antioxidant potential, selenium, polyphenols, lignans, and phytosterols did not include all foods and dishes, so we used similar products to calculate any missing data.

The strength of this study is that a new index was used to assess the antioxidant power of the diet. Moreover, standardized questionnaires were used to assess the QoL of young women with AV. Furthermore, the relationship between QoL and dietary antioxidants is rarely studied. 

## 5. Conclusions

The results of this study showed that the QoL of young women with AV is impaired. However, greater adherence to an antioxidant diet reduces the risk of AV impact on QoL by approximately 30–32% and the risk of depression by 33%. The DAQI may be used as a new indicator of diet quality in acne vulgaris. 

## Figures and Tables

**Table 1:** Characteristics of tests for assessing quality of life.

Tests Assessing the QoL	Points	Outcomes
CADI [21,22] (Cardiff Acne Disability Index)	0	No impairment
	1–5	Mild impairment
	6–10	Moderate impairment
	11–15	Severe impairment
DLQI [23] (Dermatology Life Quality Index)	0–1	No effect
	2–5	Small effect
	6–10	Moderate effect
	11–20	Very large effect
	21–30	Extremely large effect
SWLS [24] (Satisfaction With Life Scale)	5–17	Low satisfaction
	18–23	Average satisfaction
	24–35	High satisfaction
BDI [25] (Beck Depression Inventory)	0–13	No depression
	14–19	Mild depression
	20–28	Moderate depression
	29–63	Severe depression

**Table 2:** The cut-off values of the dietary components of DAQI.

Dietary Components	Score 1	Score 0
DTAC/1000 kcal [mmol]	≥Me	<Me
DP/1000 kcal [mg]	≥Me	<Me
DPH/1000 kcal	≥Me	<Me
DL/1000 kcal	≥Me	<Me
β-carotene/1000 kcal [µg]	≥Me	<Me
Vitamin E [mg]	≥90% AI (≥7.2)	<90% AI (<7.2)
Vitamin C [mg]	≥90% RDA (≥67.5)	<90% RDA (<67.5)
Iron [mg]	≥90% RDA (≥16.2)	<90% RDA (<16.2)
Zinc [mg]	≥90% RDA (≥7.2)	<90% RDA (<7.2)
Copper [mg]	≥90% RDA (≥0.81)	<90% RDA (<0.81)
Manganese [mg]	≥90% AI (≥1.62)	<90% AI (<1.62)
Selenium [µg]	≥90% RDA (≥49.5)	<90% RDA (<49.5)
Overall score	Minimum 12	Maximum 0

Me—median, RDA—recommended dietary allowances, AI—adequate intake, DL—dietary lignans, DTAC—dietary total antioxidant capacity, DP—dietary polyphenols, DPH—dietary phytosterols.

**Table 3:** General characteristics of the studied group with acne.

Variable	Women
Age (year), X ± SD (range)	23.65 ± 6.24 (18–35)
BMI (kg/m^2^), X ± SD (range)	24.35 ± 6.22
BMI, N (%)	
Underweight (<18.5 kg/m^2^)	18 (10.91)
Normal (18.5–24.9 kg/m^2^)	124 (75.15)
Overweight (25.0–29.9 kg/m^2^)	23 (13.94)
Level of education, N (%)	
Higher	48 (29.09)
Middle	107 (64.85)
Bellow middle	10 (6.06)
Working status, N (%)	
Working	37 (22.42)
Learning	126 (76.36)
Not working and learning	2 (1.22)
Marital status, N (%)	
Married/cohabiting	20 (12.12)
Single	145 (87.88)
Physical activity, N (%)	
High level	3 (1.81)
Moderate level	90 (54.55)
Low level	72 (43.64)
Smoking, N (%)	43 (26.06)
Alcohol intake, N (%)	
More than once a week	27 (16.36)
Once a week	42 (25.45)
Occasionally or never	96 (58.19)
Acne duration (years), N (%)	
<2	33 (20.00)
2–5	87 (52.73)
>5	45 (27.27)
Acne severity, N (%)	
Mild	61 (36.97)
Moderate	89 (53.94)
Severe	15 (9.09)

X—mean; SD—standard deviation; N—number; BMI—body mass index.

**Table 4:** Tests assessing the quality of life.

QoL Scores	Results
CADI, X ± SD	8.14 ± 4.25
No impairment, N (%)	45 (27.27)
Mild impairment, N (%)	48 (29.09)
Moderate impairment, N (%)	67 (40.61)
Severe impairment, N (%)	5 (3.03)
DLQI, X ± SD	9.52 ± 4.36
No effect, N (%)	26 (15.76)
Small effect, N (%)	43 (26.06)
Moderate effect, N (%)	78 (47.27)
Very large effect, N (%)	15 (9.09)
Extremely large effect, N (%)	3 (1.82)
SWLS, X ± SD	20.32 ± 4.56
Low satisfaction, N (%)	46 (27.88)
Average satisfaction, N (%)	66 (40.00)
High satisfaction, N (%)	53 (32.12)
BDI, X ± SD	9.75 ± 6.31
No depression, N (%)	115 (69.70)
Mild depression, N (%)	42 (25.45)
Moderate depression, N (%)	5 (3.03)
Severe depression, N (%)	3 (1.82)

X—mean; SD—standard deviation; N—number; QoL—quality of life; SWLS—satisfaction with life scale; CADI—Cardiff acne disability index; BDI—Beck depression inventory; DLQI—dermatology life quality index.

**Table 5:** Calculation of DAQI.

No.	Variables	X ± SD	Range	≥Me, N (%)
	Energy of diet (kcal/d.)	1625.7 ± 583.4	577.4–4556.6	-
1.	DTAC (mmol/d.)	10.8 ± 7.2	4.5–34.8	-
	DTAC/1000 kcal	6.5 ± 4.1	-	39 (23.6)
2.	DP (mg/d.)	1715.7 ± 583.2	543.2–3455.4	-
	DP/1000 kcal	993.8 ± 312.5	-	45 (27.3)
3.	DPH (mg/d.)	223.5 ± 194.3	22.4–623.5	-
	DPH/1000 kcal	137.2 ± 119.5	-	51 (30.9)
4.	DL (µg/d.)	784.7 ± 545.2	27.8–1458.4	-
	DL/1000 kcal	483.1 ± 335.4	-	49 (29.7)
5.	β-carotene (µg/d.)	3462.4 ± 2345.2	135.2–6556.7	-
	β-carotene/1000 kcal	2130.5 ± 1376.5	-	87 (52.7)
	**Variables**	**X ± SD**	**Range**	**≥90% RDA, N (%)**
6.	Vitamin C (mg/d.)	56.5 ± 36.2	5.2–267.5	42 (25.5)
7.	Zinc (mg/d.)	4.8 ± 3.5	1.8–21.4	53 (32.1)
8.	Iron (mg/d.)	7.4 ± 5.5	4.2–28.9	56 (33.9)
9.	Copper (mg/d.)	0.7 ± 0.4	0.2–3.5	68 (41.2)
10.	Selenium (µg/d.)	33.5 ± 19.3	11.5–154.3	70 (42.4)
	**Variables**	**X ± SD**	**Range**	**≥90% AI, N (%)**
11.	Vitamin E (mg/d.)	6.2 ± 4.48	1.65–28.4	51 (30.9)
12.	Manganese (mg/d.)	1.4 ± 1.2	0.4–8.5	72 (43.6)
	**DAQI, X ± SD (range)**	**8.2 ± 4.8 (3–12)**		

Me—mediane; X—mean; SD—standard deviation; N—number; RDA—recommended dietary allowances; AI—adequate intake; DTAC—dietary total antioxidant capacity; DP—dietary polyphenols; DPH—dietary phytosterols; DL—dietary lignans; DAQI—dietary antioxidant quality index.

**Table 6:** Characteristics of the study population based on DAQI tertiles.

	DAQI			*p*-Value
	T1 (1–4)	T2 (5–8)	T3 (9–12)	
Number of participants	42	85	38	
Age (years), X ± SD	22.25 ± 6.12	23.27 ± 6.85	24.62 ± 6.24	0.011
BMI (kg/m^2^)	25.45 ± 5.43	24.11 ± 5.47	22.67 ± 4.23	0.009
Level of education, N (%)				
Higher	15 (35.71)	20 (23.53)	13 (34.21)	0.545
Middle	22 (52.38)	60 (70.59)	25 (65.79)	
Bellow middle	5 (11.91)	5 (5.88)	0 (0.00)	
Working status, N (%)				
Working	10 (23.81)	15 (17.65)	12 (31.58)	0.684
Learning	32 (76.19)	68 (80.00)	26 (68.42)	
Not working and learning	0 (0.00)	2 (2.35)	0 (0.00)	
Marital status, N (%)				
Married/cohabiting	5 (11.90)	10 (11.76)	5 (13.16)	0.828
Single	37 (88.10)	75 (88.24)	33 (86.84)	
Energy of diet (kcal/d.), X ± SD	1545.2 ± 534.1	1694.2 ± 545.8	1843.2 ± 634.5	<0.001
Physical activity, N (%)				
High	0 (0.00)	0 (0.00)	3 (7.89)	<0.001
Moderate	17 (40.48)	47 (55.29)	26 (68.43)	
Low	25 (59.52)	38 (44.71)	9 (23.68)	
Smoking, N (%)	20 (47.62)	18 (21.18)	5 (13.16)	<0.001
Alcohol intake, N (%)				
More than once a week	7 (16.67)	14 (16.47)	6 (15.79)	0.576
Once a week	11 (26.19)	21 (24.71)	10 (26.32)	
Occasionally	24 (57.14)	50 (58.82)	22 (57.89)	
Acne severity, N (%)				
Mild	15 (35.71)	31 (36.47)	15 (39.47)	0.024
Moderate	22 (52.38)	47 (55.29)	20 (52.63)	
Severe	5 (11.91)	7 (8.24)	3 (7.90)	

X—mean; SD—standard deviation; T—tertile; N—number; BMI—body mass index; DAQI—dietary antioxidant quality index.

**Table 7:** Association between DAQI and QoL.

	DAQI		
	T1	T2	T3
CADI	9.72 ± 5.28	8.43 ± 4.24	7.12 ± 4.81
	*p* < 0.001		
CADI > 5 points			
Model 1 [OR (95% CI)] ^1^	1	0.64 (0.52–1.93)	0.59 (0.44–0.95) *
Model 2 [OR (95% CI)] ^2^	1	0.67 (0.57–1.85)	0.62 (0.45–0.97) *
Model 3 [OR (95% CI)] ^3^	1	0.68 (0.55–1.77)	0.70 (0.42–0.98) *
DLQI	10.75 ± 6.12	9.29 ± 5.67	7.94 ± 4.83
	*p* < 0.001		
DLQI > 5 points			
Model 1 [OR (95% CI)] ^1^	1	0.64 (0.54–1.15)	0.61 (0.45–0.96) *
Model 2 [OR (95% CI)] ^2^	1	0.65 (0.53–1.23)	0.63 (0.42–0.95) *
Model 3 [OR (95% CI)] ^3^	1	0.67 (0.55–1.26)	0.68 (0.46–0.96) *
SWLS	18.24 ± 5.38	21.2 ± 4.93	23.45 ± 5.83
	*p* < 0.001		
SWLS < 18 points			
Model 1 [OR (95% CI)] ^1^	1	0.83 (0.57–1.42)	0.94 (0.65–1.69)
Model 2 [OR (95% CI)] ^2^	1	0.89 (0.49–1.57)	0.92 (0.62–1.72)
Model 3 [OR (95% CI)] ^3^	1	0.96 (0.64–1.68)	0.95 (0.74–1.77)
BDI	7.42 ± 4.35	10.21 ± 5.17	13.12 ± 5.85
	*p* < 0.001		
BDI > 13 points			
Model 1 [OR (95% CI)] ^1^	1	0.59 (0.45–0.95) *	0.61 (0.45–0.96) *
Model 2 [OR (95% CI)] ^2^	1	0.54 (0.46–1.13)	0.63 (0.41–0.95) *
Model 3 [OR (95% CI)] ^3^	1	0.52 (0.48–1.15)	0.67 (0.42–0.97) *

OR—odds ratio; CI—confidence interval; ^1^—crude data, ^2^—data adjusted for age and daily energy intake, ^3^—data adjusted for variables in Model 2 and for BMI, smoking, level of physical activity and severity of acne; *—*p* < 0.05.

## Data Availability

The data presented in this study are available on request from the corresponding author due to due to the protection of personal data of research participants.
